# A Bivalent Bacterium-like Particles-Based Vaccine Induced Potent Immune Responses against the Sudan Virus and Ebola Virus in Mice

**DOI:** 10.1155/2023/9248581

**Published:** 2023-04-24

**Authors:** Shengnan Xu, Wujian Li, Cuicui Jiao, Zengguo Cao, Fangfang Wu, Feihu Yan, Hualei Wang, Na Feng, Yongkun Zhao, Songtao Yang, Jianzhong Wang, Xianzhu Xia

**Affiliations:** ^1^College of Veterinary Medicine, Jilin Agricultural University, Changchun, China; ^2^Changchun Veterinary Research Institute, Chinese Academy of Agricultural Sciences, Changchun, China; ^3^Shandong Agricultural University, Taian, China; ^4^College of Veterinary Medicine, Jilin University, Changchun, China; ^5^Collaborative Innovation Center for Healthy Sheep Breeding and Zoonoses Prevention and Control, Shihezi University, Shihezi, Xinjiang, China

## Abstract

Ebola virus disease (EVD) is an acute viral hemorrhagic fever disease causing thousands of deaths. The large Ebola outbreak in 2014–2016 posed significant threats to global public health, requiring the development of multiple medical measures for disease control. Sudan virus (SUDV) and Zaire virus (EBOV) are responsible for severe disease and occasional deadly outbreaks in West Africa and Middle Africa. This study shows that bivalent bacterium-like particles (BLPs)-based vaccine, SUDV-EBOV BLPs (S/ZBLP + 2 + P), generated by mixing SUDV-BLPs and EBOV-BLPs at a 1 : 1 ratio, is immunogenic in mice. The SUDV-EBOV BLPs induced potent immune responses against SUDV and EBOV and elicited both T-helper 1 (Th1) and T-helper 2 (Th2) immune responses. The results indicated that SUDV-EBOV BLPs-based vaccine has the potential to be a promising candidate against SUDV and EBOV infections and provide a strategy to develop universal vaccines for EVD.

## 1. Introduction

Ebolaviruses, members of the Filoviridae family, which are the zoonotic agents of Ebola virus disease (EVD), also known as Ebola hemorrhagic fever, which is endemic to West Africa and Middle Africa, led to more than 34000 human cases and 15,000 deaths [1]. The Ebolavirus genus consists of six antigenically distinct species, including Zaire virus (EBOV), Bundibugyo virus (BDBV), Sudan virus (SUDV), Tai Forest virus (TAFV), Reston virus (RESTV), and Bombali virus (BOMV) [2–4]. EBOV, SUDV, and BDBV are clinically relevant viruses known to cause lethal human disease, with case fatality rates up to 90%, posing a significant health threat and highlighting the urgent need for developing medical countermeasures [5, 6]. SUDV and EBOV are responsible for a large number of EVD outbreaks. SUDV has emerged seven times since its discovery in 1976 and has caused 779 cases and 412 deaths, with an average fatality rate of 53% [1]. EBOV was associated with the 2014–2016 outbreak, the largest Ebola outbreak to date, resulting in more than 28600 cases and 11300 deaths and continued to threaten global public health. On 23rd April 2022, the Ministry of Health of the Democratic Republic of the Congo declared an outbreak of EVD, and before this outbreak, the country had reported 13 EVD outbreaks since 1976 [7]. The importation of EVD cases into Europe, Asia, and the Americas in recent years calls for developing a vaccine that can induce neutralizing antibodies and thus control disease transmission.

Ebolaviruses are single-stranded negative-sense RNA viruses, whose genome is 18.9 kb, with eight subgenomic mRNAs encoding seven structural proteins in total [8]; the sequence alignment results showed that the difference in nucleotide and amino acid of Ebola virus was concentrated in glycoprotein (GP) and nucleoprotein (NP) [3]. The GP amino-acid sequences of SUDV and BDBV differ from that of EBOV by 50% and 30%, respectively [9]. The GP, the sole structure protein expressed on the virion surface, comprises two disulfide-linked furin cleavage fragments-GP1 and GP2 [10]. The membrane-associated protein is responsible for viral attachment to target cells, viral entry into cells, membrane fusion, and eliciting a protective antibody response. Thus, GP is the primary target for developing preventative vaccines [2, 11].

At present, the leading Ebola vaccines are virus-vector vaccines [12]. A recombinant vesicular stomatitis virus (VSV)-based vaccine expressing EBOV GP is the only vaccine approved by US Food and Drug Administration (FDA) [13, 14]. These virus-vector vaccines have proven to be immunogenic and protective in nonhuman primates (NHPs) and humans. Simultaneously, these vaccines elicited robust antibody responses and cell-mediated immunity responses [15–20]. The possibility of enhanced virulence when attenuated, preexisting antibodies against virus vectors, and safety problems in immunocompromised populations have limited the application of virus-vector vaccines [12, 21]. Additionally, approved by the FDA, vaccines and even therapeutic antibodies for EVD only target one species, EBOV [22–24]. Although multiple vaccine candidates and therapeutic antibodies have been developed using a variety of platforms for disease control, the emergence of a new species, such as BOMV, has raised substantial concerns due to the unpredictability of the nature of EVD outbreaks in the future, the cross-geographies transmissibility, and the ability to escape current vaccines and antibodies [4, 25, 26]. Thus, a safe and effective vaccine that can induce a potent immune response against two or more ebolaviruses is urgently needed.

The BLPs vaccines can offer safer and more effective protection to immunized subjects from pathogens. BLPs, a novel surface display system for proteins, have been widely used in developing subunit vaccines. The BLPs surface display system is based on nonliving particles derived from nongenetically modified food-grade gram-positive *Lactococcus lactis* (*L. lactis*) and a protein anchor (PA). The Generally Recognized as Safe (GRAS) background of *L. lactis* makes it highly suitable for vaccine. The PA comprises three lysin motifs (LysM) derived from the C-terminal peptidoglycan-binding domain of AcmA, an autolysin from *L. lactis* [27, 28]. A previous study showed that three LysM domains in fusion proteins have optimal peptidoglycan-binding activities and biological functions [29]. In addition, peptidoglycan, the main component of BLP particles, is a known TLR2 agonist, which can activate the innate immune system by interacting with TLRs, enhance the ability to kill and clear pathogens, and act as an adjuvant [30]. BLPs enhance the maturation and antigen-presenting capacity of host dendritic cells (DCs) by activating the TLR2 signaling pathway and increasing the expression of surface molecules, such as CD40 and MHC-II and the ability to secrete Th1-type cytokines such as TNF-*α* and IFN-*γ* [31–33]. Thus, BLPs are an excellent antigen delivery platform.

In our previous studies, BLPs vaccines for Middle East Respiratory Syndrome (MERS), Rift Valley fever (RVF), Sudan virus disease (SVD), and Zika were generated and induced both cellular and humoral immune responses in mice [29, 34–36]. Here, we prepare an EBOV-BLPs subunit vaccine that displays the ectodomain of EBOV GP on the surface of BLPs by PA. Then, we describe a bivalent bacterium-like particle-based vaccine against SUDV and EBOV, SUDV-EBOV BLPs, generated by mixing SUDV-BLPs and EBOV-BLPs at a 1 : 1 ratio and formulated with ISA 201VG plus poly (I : C) compound adjuvant. SUDV-EBOV BLPs induced potent immune responses against SUDV and EBOV in BALB/c mice and have the potential as a vaccine candidate to control the EVD spread.

## 2. Materials and Methods

### 2.1. Bacteria and Cell Culture

*Lactococcus lactis* MG1363 was cultured in M17 medium (Qingdao Hope Bio-Technology Co., Ltd., China) supplemented with 0.5% glucose (GM17) (Thermo Fisher Scientific, United States) at 30°C. Spodoptera frugiperda (Sf9; Gibco, Grand Island, NY, USA) insect cells were maintained in SFM 900 II medium (Life Technology, United States) at 27°C. Huh7 cells were maintained in DMEM (Life Technologies, United States) supplemented with 10% FBS at 37°C.

### 2.2. Construction and Expression of Recombinant Baculoviruses

**Figure 1:**
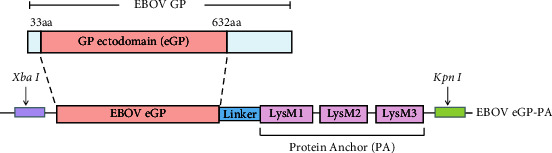
Schematic view of EBOV eGP-PA with the GP ectodomain of EBOV, linker, and PA.

The GP gene sequence of the EBOV Makona strain was retrieved from GenBank (GenBank: KJ660346.2), optimized according to the codon usage bias for insect cells, and synthesized by Sangon Biotech (Shanghai, China). Two pairs of primers were designed using Primer Premier 5.0 software (Table 1) to amplify the fusion gene fragment of the EBOV eGP-PA by overlapping PCR, in which arranged ectodomain of EBOV GP, a linker, and PA from the N-terminus to the C-terminus (Figure 1). The amplified fragment was digested with *Xba* I and *Kpn* I and then cloned into the double enzyme-digested pFastBac1 vector (Invitrogen-Life Technologies, United States) to generate the recombinant plasmid pFB-EBOV eGP-PA. Finally, pFB-EBOV eGP-PA was transformed to DH10Bac to generate the recombinant bacmid rBacmid-EBOV eGP-PA. Following the Bac-to-Bac Expression Systems manual, the recombinant bacmid was transfected into sf9 insect cells using Cellfectin II Reagent (Life Technologies, United States). The supernatants containing recombinant baculovirus rBV-EBOV eGP-PA were harvested 5 d after transfection as viral stocks. GP-PA exit was verified by PCR with oligonucleotide primers EBOV-eGP-F and Linker-PA-R from the third-generation (P3) recombinant baculovirus genome.

### 2.3. Identification of the Expression of EBOV eGP-PA Fusion Protein

The immunofluorescence analysis (IFA) was performed to confirm the expression of EBOV eGP-PA by recombinant baculovirus, as previously described [29]. Briefly, sf9 cells were infected with rBV-EBOV eGP-PA. After 48 h, the cells were fixed with 80% cold acetone for 30 min at room temperature. Following three washes with PBST, mouse anti-EBOV GP monoclonal antibody (1 : 200, prepared and stored in our laboratory only reacts specifically with SUDV GP) was added and incubated for 1 h at room temperature [36]. After three washes with PBST, a FITC-labeled goat antimouse IgG antibody (1 : 200, BioWorld, USA) was added with Evans blue (Sigma̶Aldrich, United States) and incubated for 1 h at 37°C. After washing, the cells were observed with a fluorescence microscope.

Western blotting (WB) was used to analyze the expression of the fusion protein. The sf9 cells were infected with rBV-EBOV eGP-PA at a volume ratio of 1% and then incubated for 4 d at 27°C. The cell pellets and supernatant were harvested by centrifugation at 12,000 rpm at 4°C for 10 min. Cell pellets were resuspended in PBS following three washes and placed on ice for sonication. Samples were transferred onto a polyvinylidene fluoride (PVDF) membrane (Merck Millipore, United States) after SDS-PAGE under denaturing conditions for western blotting with mouse anti-EBOV GP antibody and a horseradish peroxide (HRP)-conjugated goat antimouse antibody.

### 2.4. Preparation of BLPs and EBOV-BLPs Complexes

BLPs were prepared as previously described [37]. Briefly, the cells from the *L. lactis* strain MG 1363 were washed with PBS and boiled in 10% trichloroacetic acid (TCA) for 30 min to generate BLPs. One unit (U) was defined as 2.5 × 10^9^ BLPs and was added into 10 mL of recombinant baculovirus culture supernatant containing EBOV eGP-PA and then mixed for 30 min at room temperature. Finally, the resulting EBOV-BLPs were concentrated at 6,000×*g* for 10 min at 4°C, washed and resuspended in sterile PBS, and stored at −20°C.

### 2.5. Identification of EBOV-BLPs Complexes

The IFA, western blotting, and transmission electron microscopy (TEM) were performed to confirm the binding of the fusion protein to BLPs.

For IFA, 100 *μ*l EBOV-BLPs samples were concentrated and resuspended in 3% BSA to block for 30 min at room temperature. The mouse anti-EBOV GP monoclonal antibody and FITC-labeled goat antimouse IgG antibody were added and incubated as described above. After washing, the cells were observed with a fluorescence microscope.

For WB analysis, the pellets and supernatant from the EBOV-BLPs were mixed with 5× SDS-PAGE sample buffer (Beyotime Biotechnology, Shanghai, China), respectively. Finally, the complexes were separated by 12% SDS-PAGE and transferred onto PVDF membranes under denaturing conditions for western blotting with mouse anti-EBOV GP antibody and goat antimouse IgG antibody.

The EBOV-BLPs were prefixed with 2.5% glutaraldehyde in 0.1 M PBS (pH 7.4) at 4°C overnight for TEM. The GEM particles were used as a control. Ultrathin sections (70 nm) were stained with 2% uranyl acetate in 70% ethanol and Reynold's lead solution and examined with a JEM 1200EXII electron microscope (JEOL, Japan).

To analyze the maximum binding capacity of the fusion protein to BLPs, 1U BLPs were bound with 6 ml, 8 ml, 12 ml, and 16 ml EBOV eGP-PA fusion protein, respectively. The complexes were concentrated and resuspended in sterile PBS for western blotting to analyze the binding capacity of the fusion protein to BLPs with Gel Image System analysis software, version 4.2 (Tanon, Shanghai, China). A standard curve was obtained by SDS-PAGE using serially diluted BSA as standard with Quantity One image analysis software to determine the maximum binding amount of EBOV eGP-PA to BLPs.

### 2.6. Immunizations of Mice and the Associated Ethics Statement

Female BALB/c mice aged 6–8 weeks were randomly divided into 5 groups (*n* = 12/group) and vaccinated intramuscularly (i.m.) with the vaccines, as shown in Table 2. All BALB/c mice are handled in compliance with the guidelines for the Welfare and Ethics of Laboratory Animals of China (GB/T 35823-2018) and all protocols approved by the Animal Welfare and Ethics Committee of the Veterinary Institute at the Changchun Veterinary Research Institute (Laboratory Animal Care and Use Committee Authorization, permit number (JSY-DW-2018-02).

The SUDV-BLPs or EBOV-BLPs were mixed with a complex adjuvant of ISA 201 VG (Seppic, Paris, France) and poly (I : C) (Sigma, St. Louis, MN, USA) based on our previous research [36]. The mice in the control group received the same volume of PBS simultaneously. The prime and boost immunizations were given three weeks intervals. Sera samples were collected 2, 4, and 5 weeks after prime immunization. Spleens were collected 8 d after boost immunization.

### 2.7. Pseudovirion Neutralization Assay

The human immunodeficiency virus-based pseudovirion containing the SUDV GP or EBOV GP was generated as previously described [38]. Briefly, 293 T cells were cotransfected with the Env-defective HIV backbone plasmid, pNL4-3.Luc.RE, and the plasmid pcDNA4.0-SUDV-GP or pcDNA4.0-EBOV-GP contained the SUDV GP or EBOV GP genes, respectively. After incubation at 37°C and 5% CO_2_ for 48 h, supernatants were harvested by centrifuging at 4°C and stored at −80°C. The Huh7 cells were used to titer the pseudovirion, which was serially diluted using DMEM. The TCID_50_ of pseudovirion was calculated by the Reed̶Muench method.

The pseudovirion-neutralizing assays of SUDV and EBOV were performed as previously described [36]. Briefly, 100 × TCID_50_ pseudovirion, based on the HIV lentiviral packaging system, was mixed with an equal volume of serially diluted mouse sera, incubated for 1 h at 37°C, and then incubated with Huh 7 cells for 5 h. Each sample was performed in quadruplicate. The medium was replaced with DMEM supplemented with 10% FBS and incubated for 48 h at 37°C. The luciferase activity of the sample was measured with an Infinite M200 Microplate Spectrophotometer (Tecan, Männedorf, Switzerland).

### 2.8. Analysis of Antibody Titers by Indirect ELISA

SUDV and EBOV GP proteins were expressed and purified as previously described [39]. Sera samples from the immunized mice were collected and tested for SUDV GP- and EBOV GP-specific IgG, IgG1, and IgG2a antibodies by indirect ELISA, as described previously [36]. Briefly, 96-well microtiter plates were coated overnight at 4°C with 20 *μ*g/ml purified SUDV GP or EBOV GP (100 *μ*l/well) and then blocked for 1.5 h at 37°C after washing three times with PBST. The sera samples, twofold serially diluted in 5% skim milk, were incubated for 1 h at 37°C. Following washing, the plates were incubated with HRP-labeled goat antimouse IgG (1 : 10,000, Bioworld, United States), HRP-labeled goat antimouse IgG1 (1 : 5,000, Southern Biotechnology, United States), and HRP-labeled goat antimouse IgG2a (1 : 5000, Southern Biotechnology, United States) for 1 h at 37°C, respectively. Following washing, 100 *μ*l/well tetramethylbenzidine (TMB) substrate (Sigma-Aldrich, United States) was added. The reaction was stopped with 2 M H_2_SO_4_, and the absorbance was read at 450 nm using an automated ELISA plate reader (Thermo Fisher Scientific, United States).

### 2.9. Splenocyte Proliferation Assay

Splenocyte proliferation assay was performed as previously described [40]. Briefly, the splenocytes were cultured in 1640 medium (Gibco, San Diego, CA, United States) supplemented with 10% FBS 8 d after boost immunization and stimulated with or without purified EBOV GP antigen (10 *μ*g/ml) at 37°C and 5% CO_2_ for 44 h. And 10 *μ*l of CCK-8 solution (KeyGEN Biotech, Nanjing, China) was added to each well of the 96-well plate. The plates were incubated at 37°C, and 5% CO_2_ for 4 h. Finally, the plates were measured at 450 nm using an Infinite M200 Microplate Spectrophotometer. The stimulation index (SI) was calculated as follows:

(1) $$\begin{array}{c} SI={OD}_{450}\;for\;stimulated\frac{cultures}{{OD}_{450}}for\;non-stimulated\;cultures\text{.} \end{array}$$

### 2.10. IFN-*γ*, IL-4, and TNF-*α* ELISpot Assays

ELISpot IFN-*γ*, IL-4, and TNF-*α* cytokine assays were performed as described previously [36]. Splenocytes were cultured in Roswell Park Memorial Institute (RPMI) 1640 medium (Gibco, San Diego, CA, USA) supplemented with 10% FBS 8 d after boost immunization and stimulated with or without purified EBOV GP antigen (10 *μ*g/ml) for 36 h at 37°C in 5% CO_2_. According to the manufacturer's instructions, the splenocytes producing IFN-*γ*, IL-4, and TNF-*α* were measured using ELISpot kits (Mabtech AB, Stockholm, Sweden). The spot-forming cells (SFCs) were counted using an automated ELISpot reader (AID ELISPOT reader-iSpot, AID GmbH, GER).

### 2.11. ELISA Measurement of Cytokine Secretion

Splenocytes were harvested 8 d after boost immunization and then cultured at a density of 1 × 10^6^ cells/mL with stimulation as described above. After 48 h, the supernatants of the stimulated cells were collected by centrifugation (600 *g*, 10 min) and were evaluated using mouse IL-2, IL-4, IL-10, IFN-*γ*, and TNF-*α* ELISA kits (Mabtech AB, Sweden) according to the manufacturer's instructions.

### 2.12. Statistical Analysis

Statistical analysis was performed using GraphPad Prism software (GraphPad Software, La Jolla, CA, USA). Significant differences were determined by an unpaired Student's *t*-test. Data are presented as the mean ± standard error unless otherwise indicated. Statistical significance is indicated as ^*∗*^*P* < 0.05, ^*∗∗*^*P* < 0.01, ^*∗∗∗*^*P* < 0.001, and ^*∗∗∗∗*^*P* < 0.0001.

## 3. Results

### 3.1. Expression of EBOV eGP-PA Fusion Protein

**Figure 2:**
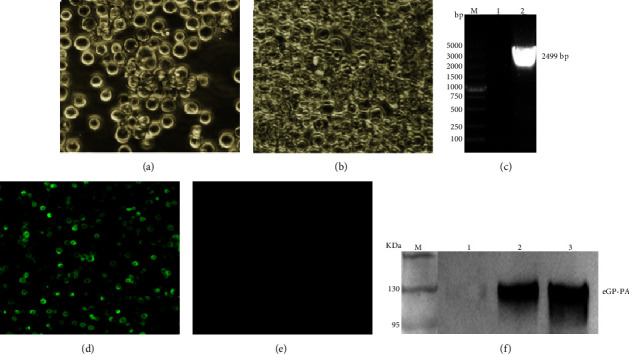
Identification of EBOV eGP-PA fusion Protein. The CPE in sf9 cells infected with rBV-EBOV eGP-PA (a) and uninfected sf9 cells (b) was observed. The PCR amplification of rBV-EBOV eGP-PA genome with oligonucleotide primers EBOV-eGP-F/Linker-PA-R (c); M: DNA marker; lane 1: control baculovirus; lane 2: the P3 recombinant baculoviruses. IFA detection of the expression of EBOV eGP-PA in baculovirus-infected Sf9 insect cells (magnification of microscopy images, 200x). The sf9 cells, fed in 24 well plates, were incubated with mouse anti-EBOV GP monoclonal antibody and then detected using conjugated goat antimouse IgG (d, e). WB analysis of EBOV eGP-PA fusion protein expression patterns (f). M: protein marker; lane 1: culture medium supernatant; lane 2: precipitate following supersonic schizoanalysis; lane 3: supernatant following supersonic schizoanalysis. GP: 100 kDa; PA: 30 kDa.

To construct pFB-EBOV eGP-PA expressing EBOV eGP-PA, the gene of EBOV eGP-PA was synthesized and amplified and then cloned into pFastBac1, which was transformed to DH10Bac to generate the rBacmid-EBOV eGP-PA. The recombinant baculovirus rBV-EBOV eGP-PA, rescued by transfection of sf9 insect cells with the rBacmid-EBOV eGP-PA, was identified by CPE, PCR, and IFA. The Sf9 cells infected with rBV-EBOV eGP-PA released from the plate appeared to have much lysis and floating (Figure 2(a)) were compared with the mock group (Figure 2(b)). Nucleic acid electrophoresis stain of the EBOV eGP-PA gene showed gene migration in the expected size of 2499 bp. It demonstrated that the EBOV eGP-PA gene was detected in the rBV-EBOV eGP-PA by PCR (Figure 2(c)). The IFA results showed that EBOV eGP-PA was expressed in sf9 insect cells infected with rBV-EBOV eGP-PA since strong green fluorescence signals were detected (Figure 2(d)), while no fluorescence signals were detected in the control group (Figure 2(e)). The WB analysis showed that a 130 kDa band corresponding to the eGP-PA3 fusion protein was detected in the supernatant and lysate after sonication samples, while no band was detected in the cells culture supernatant sample (Figure 2(f)).

### 3.2. Identification of BLPs and EBOV-BLPs

**Figure 3:**
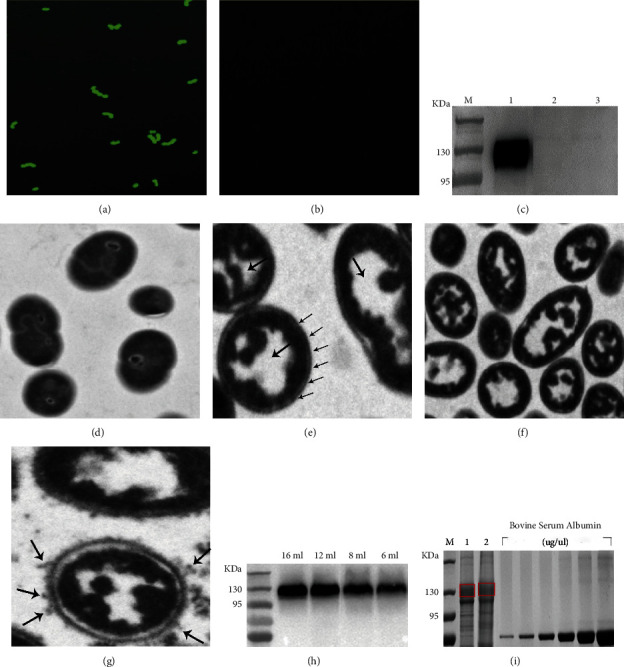
Identification of BLPs loaded with EBOV eGP-PA. IFA analysis of the BLPs loaded with EBOV eGP-PA (magnification of microscopy images, 1000x). BLPs loaded with EBOV eGP-PA (a); BLPs without fusion protein loaded (b). WB analysis of EBOV eGP-PA fusion protein binding with BLPs (c). M: protein marker; lane 1: the precipitate of fusion protein bound to BLPs; lane 2: the supernatant of fusion protein bound to BLPs; lane 3: BLPs. Transmission electron microscopy analysis of L. lactis without (d) or with TCA treated (e) and BLPs without fusion protein loaded (f) and BLPs loaded with EBOV eGP-PA fusion protein (g). WB analysis of the binding amount of the fusion protein on BLPs (h) SDS-PAGE analysis of the maximum binding capacity of EBOV eGP-PA fusion protein to BLPs (i). M: protein marker; 1: combined SUDV eGP-PA; 2: combined EBOV eGP-PA.

To analyze BLPs loaded with EBOV eGP-PA fusion protein, IFA, WB, and TEM were performed. Unlike naked BLPs, EBOV-BLPs, which reacted with a specific monoclonal antibody bound with FITC-labeled goat antimouse IgG antibody, emitted strong green fluorescence signals (Figures 3(a) and 3(b)). After binding EBOV eGP-PA fusion protein with BLPs, a 130 kDa of EBOV eGP-PA fusion protein band was detected in the lysate of EBOV-BLPs (Figure 3(c), lane 1). In contrast, no detection of protein band was identified in both supernatant and BLPs without fusion protein loaded (Figure 3(c), lane 2). Furthermore, TEM was used to analyze the difference between the naked BLPs and EBOV-BLPs loaded with EBOV eGP-PA fusion protein compared with TCA-untreated *L. lactis*. As shown in Figures 3(d) and 3(e), TCA-pretreated *L. lactis* (BLPs) maintained the original peptidoglycan backbone and morphology, with a smooth surface and hollow interior, indicating that inner protein and DNA were degraded. There were many fine floccules on the surface of the EBOV-BLPs, which further verified that the EBOV eGP-PA fusion protein was successfully anchored and displayed (Figures 3(f) and 3(g)). These data demonstrated that eGP-PA fusion protein was successfully bound with EBOV-BLPs.

### 3.3. The Maximum Binding Capacity of the Fusion Protein to BLPs

SDS-PAGE and WB were performed to confirm the maximum binding capacity of EBOV eGP-PA fusion protein to BLPs. WB results show that the maximum binding volume of EBOV eGP-PA fusion protein to 1 U BLPs was 12 ml (Figure 3(h)). After SDS-PAGE, grayscale values were calculated using Image J analysis software to obtain the standard curve (*Y* = 0.000680*X*−0.41, *R* = 0.996820) and the binding amount of EBOV eGP-PA fusion protein to 1 U BLPs was 68.9 *μ*g (Figure 3(i)).

### 3.4. Antibody Responses Induced by SUDV-EBOV BLPs

**Figure 4:**
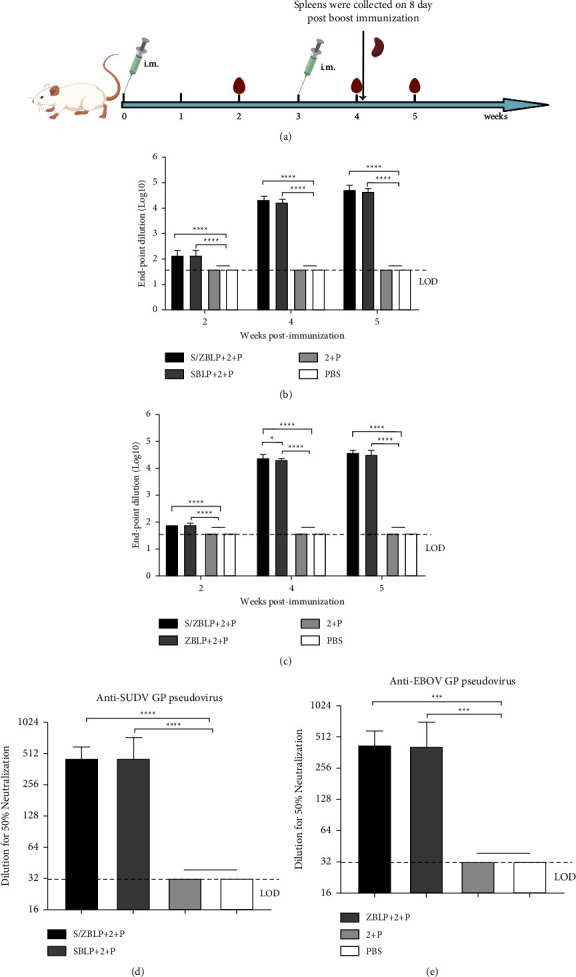
Serum antibody responses induced by SUDV-EBOV BLPs. Overall study design (a). Mice were intramuscularly immunized with S/ZBLP + 2 + P, SBLP + 2 + P, ZBLP + 2 + P, 2 + P, and PBS 3 weeks apart. Serum samples of immunized mice were collected by retro-orbital plexus puncture at 2, 4, and 5 weeks after prime immunization, respectively. To assess cell-mediated immune responses induced by SUDV-EBOV BLPs, spleens were collected 8 d after boost immunization. The horizontal dotted line in the figure indicates the limit of detection (LOD). Data are shown as the mean ± SD and were analyzed by one-way ANOVA. The SUDV-and EBOV-specific IgG antibody levels induced by SUDV-EBOV BLPs were analyzed using indirect ELISA (b, c). Neutralizing antibody levels of serum samples were determined using pseudovirion neutralization assay (d, e).

To assess humoral responses induced by the SUDV-EBOV BLPs vaccine candidate, serum samples were collected 2 weeks after prime immunization and 1 week after boost immunization, respectively (Figure 4(a)), and SUDV- and EBOV-specific IgG antibody levels were analyzed using indirect ELISA. As shown in Figures 4(b) and 4(c), SBLP + 2 + P- and EBLP + 2 + P-immunized mice exhibited induction of specific IgG antibodies against SUDV and EBOV, respectively. Importantly, immune responses against SUDV and EBOV were detected in S/EBLP + 2 + P-immunized mice. Furthermore, specific IgG antibody levels were significantly increased in all immunized groups at 1 week after boost immunization, and immunization with S/EBLP + 2 + P induced higher levels of EBOV-specific antibodies than those induced by EBLP + 2 + P. At 2 weeks after boost immunization, high levels of SUDV IgG antibodies (1 : 56320) and EBOV IgG antibodies (1 : 38400) were detected in the sera samples of mice in the S/EBLP + 2 + P-immunized group.

To test whether the serum samples of mice immunized with the SUDV-EBOV BLPs vaccine candidate could exhibit virus-neutralizing activity, neutralization assays were performed to detect neutralizing antibody levels in serum samples collected at 2 weeks after boost immunization. The results showed that neutralizing antibodies were detected in all immunized mice serum samples except for the control group. Among them, the S/EBLP + 2 + P immunization group could induce SUDV- and EBOV-specific neutralizing antibodies (Figures 4(d) and 4(e)). In the S/ZBLP + 2 + P immunization group, the same anti-SUDV specific neutralizing antibody titer was induced in the serum of mice under immunization with SBLP + 2 + P, and the average neutralizing antibody titer was 1 : 464 (Figure 4(d)). The average anti-EBOV neutralizing antibody titer in the S/EBLP + 2 + P immune group was 1 : 416, slightly higher than that in the ZBLP + 2 + P immunization group (Figure 4(e)).

### 3.5. Detection of SUDV-and EBOV-Specific IgG Subtypes

**Figure 5:**
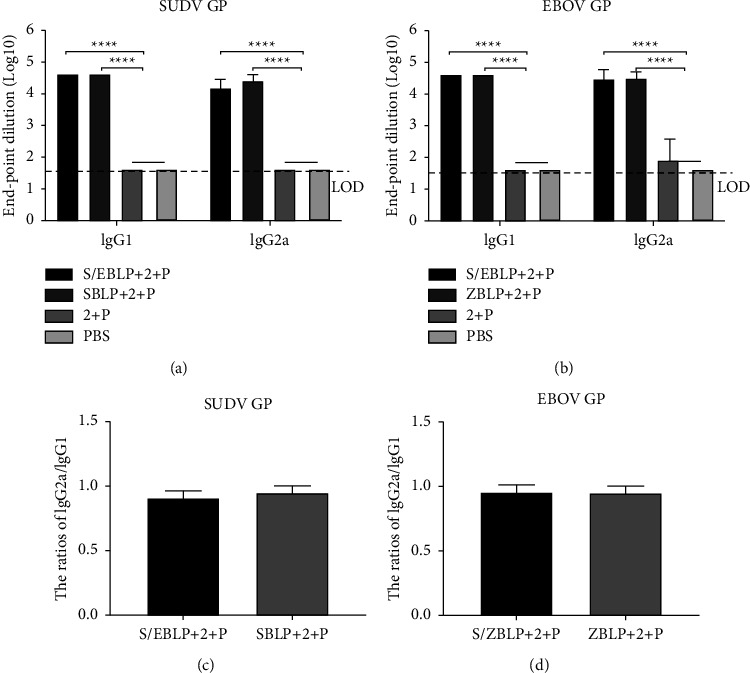
SUDV GP- and EBOV GP-specific IgG antibody subtypes responses induced by SUDV-EBOV BLPs. Serum samples were collected to detect SUDV GP- and EBOV GP-specific IgG1 and IgG2a antibody levels using indirect ELISA and IgG1, and IgG2a antibody responses were displayed as the end-point dilution titers. The ratios of IgG2a/IgG1 were calculated. Data are shown as the mean ± SD and were analyzed by one-way ANOVA. SUDV GP-IgG1 and IgG2a antibody levels (a) and IgG2a/IgG1 ratios (c) EBOV GP-specific IgG1 and IgG2a antibody levels (b) and IgG2a/IgG1 ratios (d)

To further evaluate the Th1 and Th2 balance, SUDV- and EBOV-specific IgG subtypes and the ratios of IgG2a/IgG1 were analyzed using indirect ELISA. Immunization with S/ZBLP + 2 + P, SBLP + 2 + P, and ZBLP + 2 + P induced potent IgG1 and IgG2a antibody responses, compared with 2 + P and PBS groups. The IgG2a/IgG1 in all immunized groups was approaching 1, indicating that the SUDV-EBOV BLPs vaccine candidate elicited balanced Th1-type cellular and Th2-type humoral immune responses in mice (Figure 5).

### 3.6. Splenocyte Proliferation

**Figure 6:**
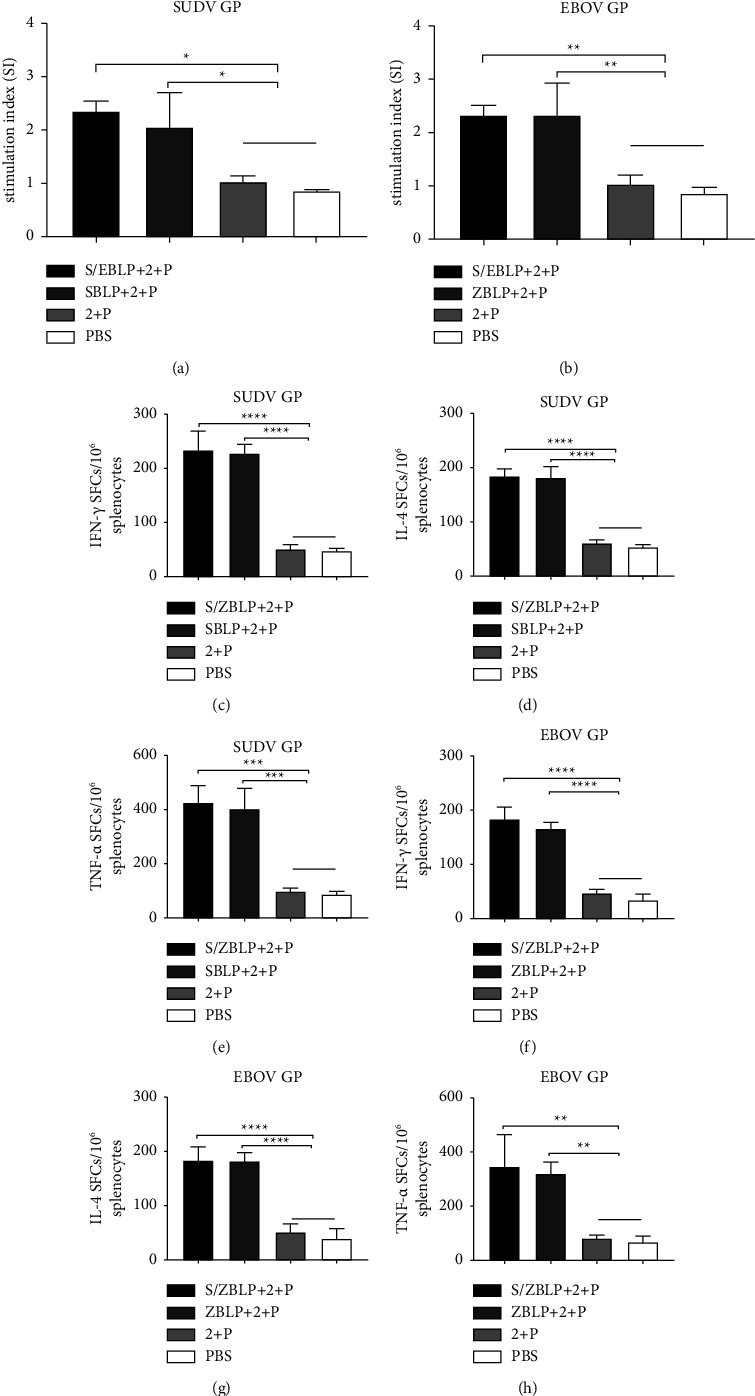
Cell-mediate responses induced by SUDV-EBOV BLPs vaccine. Data are shown as the mean ± SD and were analyzed by one-way ANOVA. The splenocytes, isolated from mice, were used to perform a splenocyte proliferation assay and detect the antigen-specific IFN-*γ*, IL-4, and TNF-*α* secretion levels using ELISpot. The stimulation index of the splenocytes was detected using CCK-8 solution by stimulating the splenocytes with purified SUDV GP (a) and EBOV GP (b), respectively. Splenocytes secreting IFN-*γ*, IL-4, and TNF-*α* were quantified using an ELISpot assay after SUDV GP (c–e) and EBOV GP (f–h) stimulation.

To further assess SUDV-EBOV BLPs vaccine candidate-induced cell-mediated responses, the splenocytes from mice in all groups were harvested 8 d after boost immunization for splenocyte proliferation assay and the detection of IFN-*γ*, IL-4, and TNF-*α* secretion. After SUDV GP and EBOV GP stimulation, all the immunized groups induced splenocyte proliferation, compared with 2 + P and PBS groups. However, no significant differences were observed between the S/ZBLP + 2 + P immunized group, the SBLP + 2 + P immunized group, or the ZBLP + 2 + P immunized group (Figures 6(a) and 6(b)).

### 3.7. Antigen-Specific Cellular Immune Responses

Furthermore, IFN-*γ*, IL-4, and TNF-*α* secretion were detected using ELISpot kits, and the differences between spot-forming cells (SFCs) with stimulants and SFCs without stimulants were analyzed. The ELISpot results showed that immunization with S/ZBLP + 2 + P, SBLP + 2 + P, and ZBLP + 2 + P induced significant SFCs, compared with 2 + P and PBS (Figures 6(c)–6(h)). These data demonstrated that the SUDV-EBOV BLPs vaccine candidates elicited Th1-type cellular and Th2-type humoral immune responses in mice.

### 3.8. Splenocyte Cytokine Secretion Induced by SUDV-EBOV BLPs

**Figure 7:**
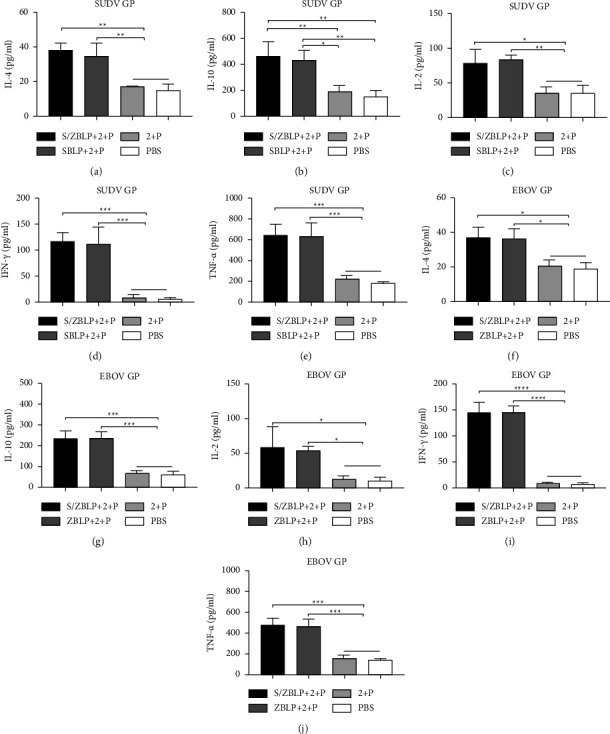
Cytokine secretion of splenocyte culture supernatant. Data are shown as the mean ± SD and were analyzed by one-way ANOVA. Cytokine secretion levels in the supernatant were measured with commercial ELISA kits. The concentration of IL-4, IL-10, IL-2, IFN-*γ*, and TNF-*α* (a–e) was measured after stimulation with SUDV GP and EBOV GP (f–j).

After stimulation with SUDV GP and EBOV GP, immunization with S/ZBLP + 2 + P, SBLP + 2 + P, and ZBLP + 2 + P induced significantly higher levels of the IL-4, IL-10, IFN-*γ*, TNF-*α*, and IL-2 cytokine secretion than that induced by 2 + P and PBS. Moreover, compared with SBLP + 2 + P and ZBLP + 2 + P immunized groups, similar IL-4, IL-10, IFN-*γ*, TNF-*α*, and IL-2 cytokine secretion levels were induced in S/ZBLP + 2 + P immunized group (Figure 7).

## 4. Discussion

Vaccine is considered an effective measure to reduce the impact of serious infectious diseases due to the induction of neutralizing antibodies, which can interfere with the invasion of viruses into cells [41]. An ideal vaccine for Ebola virus disease (EVD) should be economical, safe, and could provide protection across several species of Ebolavirus and induce long-term immunity [42]. There are six species in Ebolaviruses, and two of them, SUDV and EBOV, are highly pathogenic and responsible for many large-scale epidemics. Since the Zaire virus and Sudan virus were first reported in 1976, EVD has caused enormous public health and economic impacts in endemic countries [43]. Although SUDV and EBOV belong to the Ebolavirus genus, they are antigenically distinct due to the genome's 35% to 45% difference [44]. Thus, the monovalent EVD vaccine does not protect other filoviruses. However, most current vaccines are only indicated for one of these two viruses, including the Ervebo (rVSV-EBOV), Zabdeno/Mvabea (Ad26-ZEBOV/MVA-BN-Filo), and cAd3-EBOZ (chAd3-EBOZ) [45, 46]. Therefore, there is an urgent need to develop a bivalent and convenient EVD vaccine that protects against both EBOV and SUDV infection.

Bacterium-like particles are a novel antigen delivery system and are also a benefit for the preparation of multivalent vaccines. Multivalent vaccines can be made by attaching different antigen-PA fusions to the same particle or by combining monovalent vaccines. Compared with the virus-vectored vaccine, the BLPs delivery system has a more straightforward manufacturing process and no safety problems. Based on the BLPs surface display platform established previously, we developed BLPs-based vaccines carrying SUDV glycoprotein via the baculovirus expression system. Here, we expressed the EBOV GP ectodomain linked with PA using the same insect cell-baculovirus expression system. The results of IFA, western blotting, and TEM confirmed that the EBOV GP-PA fusion protein was anchored on the surface of BLPs. Then, a bivalent BLPs-based vaccine SUDV-EBOV BLPs was developed by mixing with the immunogenic SUDV-BLPs and EBOV-BLPs, at a 1 : 1 ratio adjuvanted with ISA 201VG plus poly (I : C) compound.

As a eukaryotic expression system, the insect cell-baculovirus expression system is a powerful platform for protein production with many advantages, such as a high density of cells, high post-translational modification levels, and the suitability for the assembly of VLPs by natural structure [47–49]. It was previously demonstrated that EBOV glycoprotein (GP) purified using a Drosophila S2 expression system can elicit active and passive protective immunity in mice when properly adjuvanted [50].

IgG subclass expression is influenced by Th1 and Th2 cytokines, such as IgG1 and IgG2a. IgG2a is the most activating effector in antiviral immunity, and the IgG2a/IgG1 reflects Th1/Th2 immune response polarization [29, 51]. In this study, the ratios of IgG2a/IgG1 were analyzed. It was shown that S/ZBLP + 2 + P elicited balanced Th1- and Th2-type immune responses in mice that were consistent with our previous results [34]. The type I interferon includes IFN*α* and IFN*β* and plays a vital role in early virus infection. Additionally, type II interferon (IFN*γ*) is critical in innate and adaptive responses [52]. Expression of type I interferon protects nonhuman primates (NHPs) from the EBOV [53]. Furthermore, we evaluated cell-mediated immunity responses by splenocyte proliferation assay and detected IL-4, IL-10, IFN-*γ*, TNF-*α*, and IL-2 cytokine secretion levels. The results showed that the SUDV-EBOV BLPs vaccine significantly induced splenocyte proliferation compared with the control group. It effectively stimulated the secretion of Th1 (IFN-*γ*, TNF-*α*, and IL-2) and Th2 (IL-4 and IL-10) cytokines in mice. The clinical symptoms and severity of EVD are associated with Ebola virus-specific T-cells, interleukins, and interferon expressions [52, 54–56]. Our data generally indicated that SUDV-EBOV BLPs have excellent immunogenicity and induced a strong antibody response and Th1/2 cytokine response in immunized mice.

In summary, we developed a bivalent BLPs-based vaccine covering the EBOV and SUDV by mixing with EBOV-BLPs and SUDV-BLPs at a 1 : 1 ratio formulated with ISA 201VG plus poly (I : C) compound adjuvant. The SUDV-EBOV BLPs induced potent and balanced immune responses against SUDV and EBOV simultaneously regarding high antibodies and cell-mediated immune response levels. ISA 201VG plus poly (I : C) compound-adjuvanted SUDV-EBOV BLPs inform the next-generation design of effective EVD vaccine candidates.

## Figures and Tables

**Table 1:** The sequence of the primers used in this study.

Primers	Sequence (5′-3′)	Products
EBOV-GP-F^1^	ATTCTGCCTTTGCGTCTAGAATCCCCCTC GGAGTCATCCACAACA	
EBOV-GP-linker-R	ACCAGAACCACCACCAGAACCACCGTCAA CGAAGTCGTGGATGATCTGGT	EBOV eGP
Linker-PA-F^2^	GGTGGTTCTGGTGGTGGTTCTGGTGAT GGTGCTTCTTCAG	
Linker-PA-R^1^	TAGTACTTCTCGACAAGCTTGGTACCTT ACTTGATACGCAGGTATTGACCGATC	PA

^1^The sequences of restriction enzyme sites are underlined and italicized. ^2^The central linker (Gly-Gly-Ser-Gly) x2 base sequences are underlined.

**Table 2:** The mice vaccination protocols.

Group	Immunization route	Antigen	Adjuvant
S/ZBLP + 2 + P	Intramuscular	10 *μ*g SUDV-BLPs + 10 *μ*g EBOV-BLPs	ISA 201 VG + poly (I : C)
SBLP + 2 + P	Intramuscular	10 *μ*g SUDV-BLPs	ISA 201 VG + poly (I : C)
ZBLP + 2 + P	Intramuscular	10 *μ*g EBOV-BLPs	ISA 201 VG + poly (I : C)
2 + P	Intramuscular	201 VG + poly (I : C)	ISA 201 VG + poly (I : C)
PBS	Intramuscular	PBS	ISA 201 VG + poly (I : C)

## Data Availability

The data used to support the finding of this study are available from the corresponding author upon request.
